# Flow imaging in vivo using off resonance spin labeling induced by extraneous contrast agent

**DOI:** 10.1186/1532-429X-18-S1-P258

**Published:** 2016-01-27

**Authors:** Jessica A Bastiaansen, Jean-Baptiste Ledoux, Andrew J Coristine, Helene Feliciano, Debora Bonvin, Marijana Mionic, Heinrich Hofmann, Matthias Stuber

**Affiliations:** 1 Department of Radiology, University Hospital (CHUV) and University of Lausanne (UNIL), Lausanne, Switzerland; 2 grid.433220.4Center for Biomedical Imaging (CIBM), Lausanne, Switzerland; 3 grid.5333.60000000121839049Powder Technology Laboratory, Materials Science and Engineering, Swiss Federal Institute of Technology, Lausanne, Switzerland

## Background

**Figure 1:**
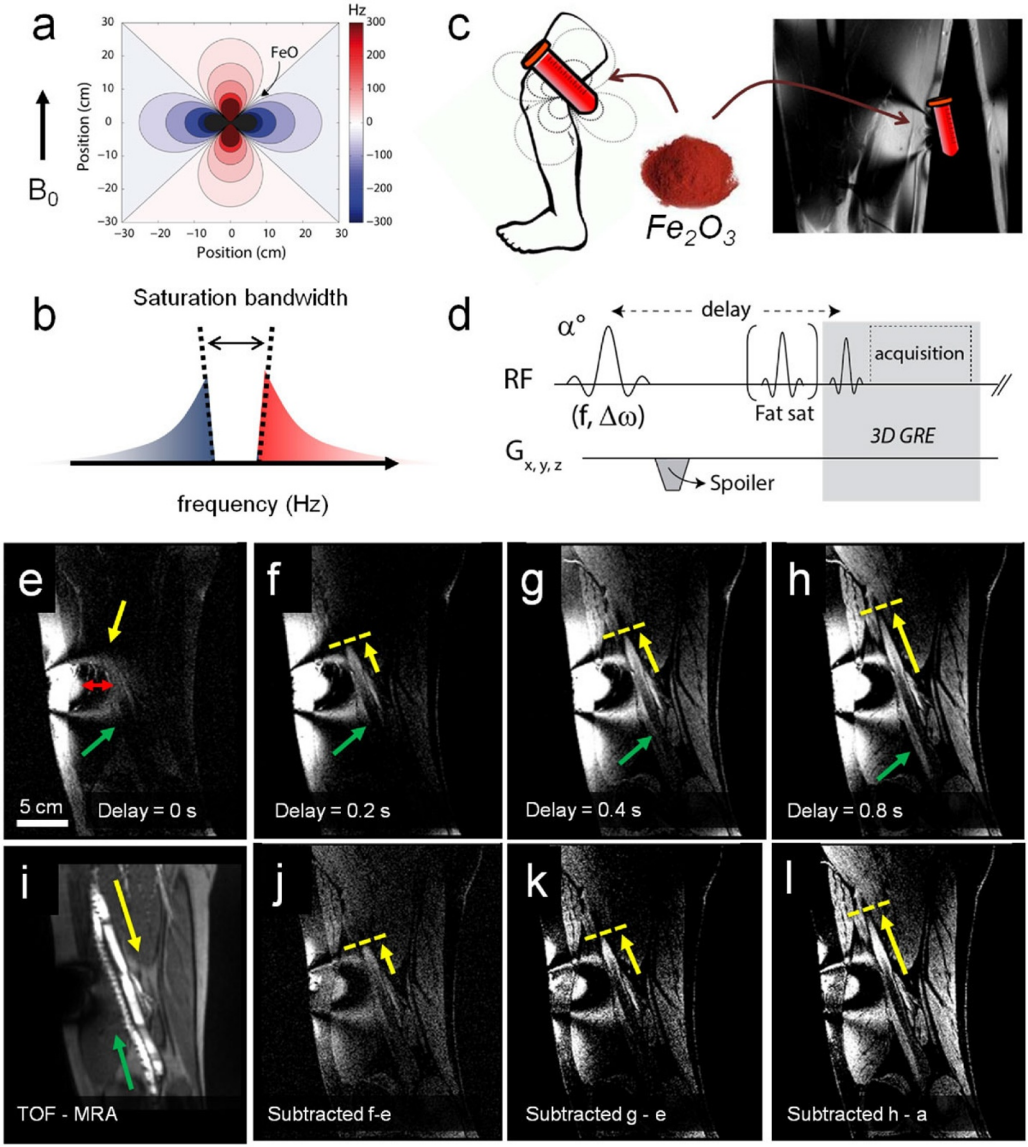
**Frequency distribution surrounding a cylindrical tube containing a suspension of iron oxide nanoparticles**. (a). Positive contrast MRI is thus enabled by the specific saturation of on-resonant spins (b). In human volunteers, a cylindrical iron oxide loaded container was placed adjacent to the upper leg causing a frequency dispersion in the tissue (c). By applying a 3D GRE MRI acquisition preceded by a narrow band saturation RF pulse with a specific delay time, flowing spins are effectively tagged (d). This tag progresses as delay times are increased from 0 ms (e) to 0.8 s (h), visualizing flow along the femoral vein (yellow arrow) and artery (green arrow). Time of flight (TOF) MRA displaying the femoral vein and artery (i). A specific off resonance frequency band (red arrow, 1e) is clearly affected by the fat saturation pulse. To enhance the moving contrast front,the images with a delay time of 0 s were subtracte from those with a delay time of 0.2 s (j), 0.4 s (k) and 0.8 s (l).

Tissues within the dipolar field of superparamagnetic contrast agents experience a frequency shift that enables positive contrast MRI with acquisition schemes using on-resonant saturation (Figure 1a,b). The administration of iron oxide based agents enables a plethora of applications using positive contrast MRI. They have been widely explored and mainly focused on lymphography, angiography, cancer detection and atherosclerosis. In this preliminary in vivo study, a dynamic contrast mechanism is presented,using externally placed suspensions of iron oxide nanoparticles (SPIONs) to induce noninvasive spin tagging of nearby blood flow.

## Methods

A cylindrical tube containing a concentrated suspension of 19 nm SPIONs was placed alongside the upper leg in human volunteers (Figure 1c), causing a local magnetic field change in the adjacent tissue. Protons residing in the off-resonance field experience a frequency shift as they move through or are static within this field. A 3D gradient echo based MRI acquisition, preceded by a narrow or broad bandwidth (100 Hz or 2 kHz) on-resonant saturation pulse (Figure 1d), was used for data acquisition on a 3T clinical MRI scanner. This led to a selective excitation of the off resonance area (Figure 1c). A variable radio frequency excitation angle and delay time ranging from 0 to 0.8 s facilitated the tagging of the off-resonant flowing spins in the nearby veins and arteries. Flowing spins remain unaffected by the narrowband RF pulse and are tagged by the external SPIONs.

## Results

**Figure 2:**
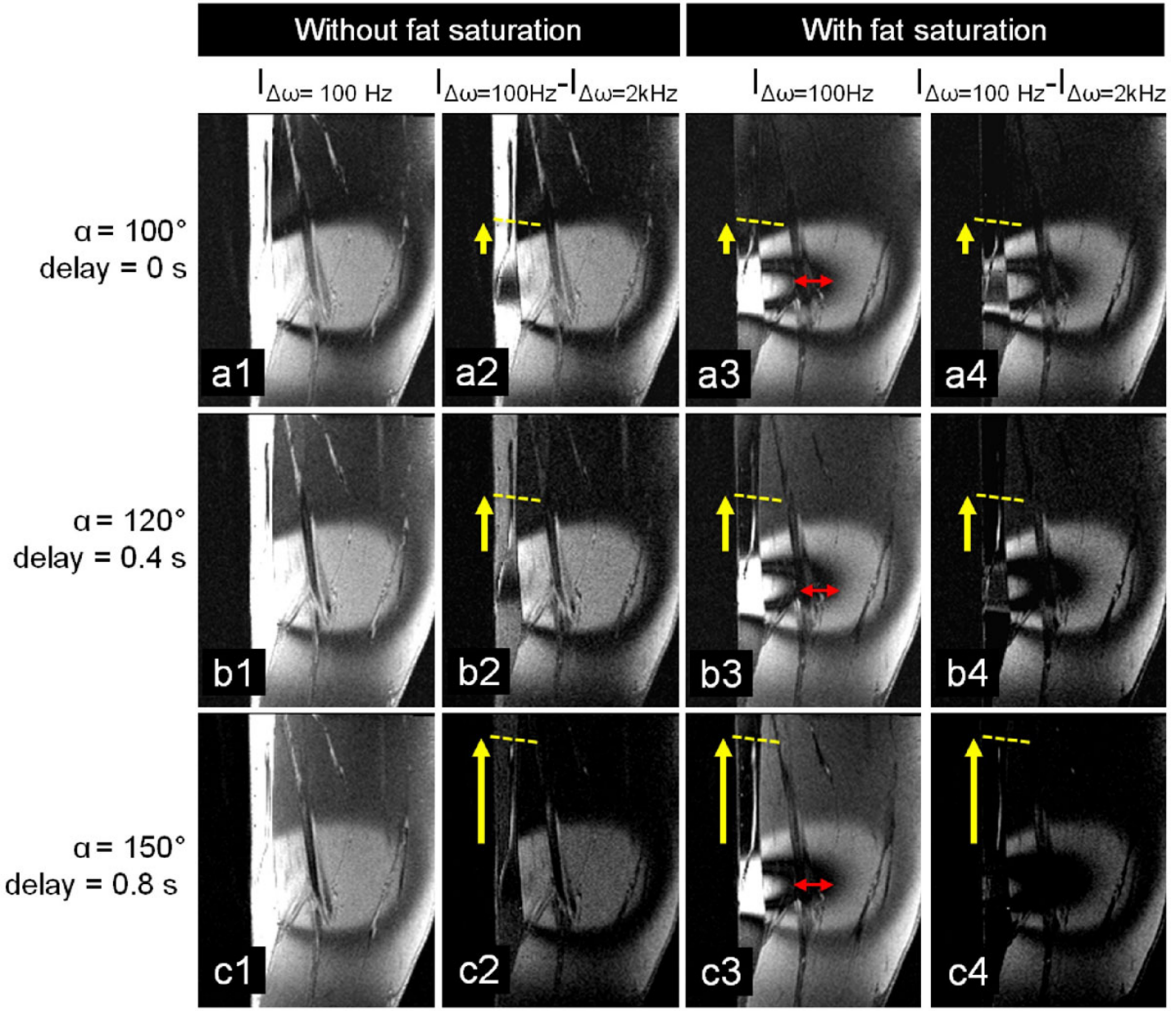
**Flowing spins within the off resonance field are positively tagged as delay times are increased from 0 s (a) to 0.4 s (b) to 0.8 s (c), visualizing flow along the femoral vein**. To compensate for T1 recovery during the delay time, 3D images acquired with a saturation bandwidth of 2 kHz were subtracted from those acquired with a saturation bandwidth of 100 Hz (a2, b2, c2), enhancing the tagged spins. The off-resonant frequency band around 400 Hz is clearly affected by the fat saturation pulse (red arrow, a3-c3). The same T1 compensation was also applied to fat suppressed images (a4-c4). Note that the fat suppression is played out before the acquisition and would not affect the flowing tagged spins relocated away from this specific fat saturation frequency band.

The off-resonance area was selectively excited and visualized with a 0 s delay time (Figure 1e). Spins were tagged up to 8 cm from the tissue surface with a 100 Hz suppression bandwidth. By extending the delay time between saturation pulse and acquisition, flowing protons that were within the SPIONs affected area still showed positive contrast in further downstream areas and their motion along the femoral vein and arteries could be tracked up to 6.7 cm showing a poiseuille shaped velocity profile (Figure 1e-h). A calculated blood velocity of 8.4 cm/s was within range of published values of 13.9 ± 5.9 cm/s. The surrounding tissue experienced magnetization recovery due to T1 and was excited with increasing RF excitation angles and delay time, in order to enhance tissue contrast. Applying a broadband RF pulse compensated for T1 recovery of surrounding tissue when subtracted from narrowband images (Figure 2). Fat saturation enhanced the positive contrast, although with unwanted saturation of a frequency band around 400 Hz, which suppressed nearly half of the off-resonant area between 3.4 cm and 6.2 cm from the skin.

## Conclusions

Flow imaging with iron induced spin tagging (FLIRT) was achieved using external SPIONs which targeted the positive labeling of moving protons. The feasibility of this method was shown in vivo with preliminary data obtained in human volunteers. It allows for flow visualization in the vicinity of ferrimagnetic objects without contrast injection.

